# Susceptibility of *Tuta absoluta* (Lepidoptera: Gelechiidae) Pupae to Soil Applied Entomopathogenic Fungal Biopesticides

**DOI:** 10.3390/insects12060515

**Published:** 2021-06-02

**Authors:** Reynardt Erasmus, Johnnie van den Berg, Hannalene du Plessis

**Affiliations:** Unit for Environmental Sciences and Management, IPM Program, North-West University, Potchefstroom 2520, South Africa; johnnie.vandenberg@nwu.ac.za (J.v.d.B.); hannalene.duplessis@nwu.ac.za (H.d.P.)

**Keywords:** biopesticide, pest management, pupae, soil application, tomato pinworm

## Abstract

**Simple Summary:**

The invasive tomato pinworm is one of the most destructive insect pests of tomato in Africa. The majority of farmers respond to infestations by applying chemical insecticides. However, the overreliance on this control method has deemed several insecticides ineffective due to resistance evolution. It is therefore crucial that integrated approaches are put in place, of which biopesticides play an important role, to mitigate this problem. Amongst the biopesticides, entomopathogenic fungi (EPF) are promising options. EPF applications aimed to control this pest have been highly effective, although the majority are applied against the larval and egg stages. This study investigated the susceptibility of pupae of the tomato pinworm against EPF products (*Beauveria bassiana* and *Metarhizium anisopliae*) when applied as a soil drench. High pupal mortality rates were recorded for all EPF products tested in bioassays and growth tunnel experiments. A significant reduction in fecundity was observed in moths that survived the pupal EPF applications, with no effects on moth fertility. Overall, our findings provide evidence of the suppressive potential when administering EPF conidia as a soil drench to disrupt the life cycle of *Tuta absoluta* for use in integrated pest management programs.

**Abstract:**

Management of *Tuta absoluta* (Meyrick) (Lepidoptera: Gelechiidae) in greenhouses and under open-field tomato cultivation relies on an integrated approach, largely targeting the egg and larval stages of the pest. However, little to no research has been done on the efficacy of EPFs for control of the pupal stage. The aims of this study were to determine the susceptibility of *T. absoluta* pupae to *Beauveria bassiana* and *Metarhizium anisopliae* spores applied as soil drench treatments, and the possible effects of these treatments on fecundity and fertility of moths. The lethal concentrations (LC_50_ and LC_80_) of the respective products were estimated in dose-response bioassays by exposing pupae in a soil substrate to different concentrations of EPF products. Emerging moths were paired in different combinations, according to EPF exposure treatments after which fecundity and fertility of females were recorded. Pupae in the soil were effectively controlled by all EPF products in both bioassays as well as in a growth tunnel experiment. The LC_50_ value of the *B. bassiana* oil formulation was significantly lower than that of other treatments. The fecundity of females that were subjected to the *B. bassiana* oil formulation was significantly lower than that of the control treatment. This study showed the potential of soil drench applications of both *B. bassiana* and *M*. *anisopliae* for control of *T. absoluta* pupae.

## 1. Introduction

*Tuta absoluta* (Meyrick) (Lepidoptera: Gelechiidae) extended its geographical distribution around the world since it was first reported outside its native range in South America, in 2006. *Tuta absoluta* was reported for the first time in the eastern parts of Spain from where it rapidly spread through Europe, the Middle East, Asia, and Africa [1,2,3,4,5,6,7,8].

The application of synthetic chemical insecticides is the most commonly used practise to control *T. absoluta* infestations in tomato crops. However, the overreliance on insecticides exerts selection pressure that favours the survival of resistant genotypes, evidently leading to the evolution of resistance and reduced efficacy of insecticides [9,10,11,12]. It is therefore crucial that an integrated approach that includes biological control, pheromone-based control methods, and biopesticides, in combination with synthetic insecticides when needed, is developed for the management of *T. absoluta* [9,13,14,15,16,17].

Biological control through the release of parasitoids and predators, as well as application of biopesticides have been investigated as management options for *T. absoluta* [18]. Amongst these, entomopathogenic fungi (EPFs) represent one of the most promising options. EPFs have been reported to be highly efficacious against a wide range of pests, especially subterranean pests [19,20]. Both *Beauveria bassiana* (Hypocreales: Cordycipitaceae) and *Metarhizium anisopliae* (Hypocreales: Clavicipitaceae) have been reported to be effective against *T. absoluta* [21,22,23]. However, these studies largely focused on the efficacy of these mycoinsecticides against the egg and larval stages of this pest. Only one study evaluated the efficacy of fungal pathogens against *T. absoluta* pupae in the soil and reported effective control with an *M. anisopliae* formulation applied as a soil drench [24].

The aims of this study were to determine the susceptibility of *T. absoluta* pupae to *B. bassiana* and *M. anisopliae* in powder and oil formulations applied as soil drench treatments, and the possible effects of these EPFs on fecundity and fertility of moths that emerge from surviving EPF-treated pupae.

## 2. Materials and Methods

### 2.1. Insect Rearing

A rearing colony was established from a *T. absoluta* population collected from infested tomato fields at Mareetsane in the North West Province of South Africa (S 26° 41′ 52″; E 25° 26′ 24″). In the laboratory, the infested leaves were placed onto a plastic mesh (hole diameter = 2 cm) which was suspended from the roof of an insect rearing cage (100 cm × 100 cm × 150 cm), above healthy potted tomato plants (cv. Monica). This allowed larvae to migrate from the sampled leaves onto the potted tomato plants to complete their life cycle. Adults were collected on a daily basis from this rearing cage and transferred to oviposition cages that contained healthy potted tomato plants. Cotton swabs soaked in a 10% sugar solution were provided in Petri dishes at the bottom of the cages, as an energy source for moths. Plants were replaced every second day to ensure that the eggs recovered from these plants were of a similar age and that they would hatch at approximately the same time. Mass rearing of this *T. absoluta* population was done for several generations as described above.

To use pupae of a similar age in bioassays, *T. absoluta* infested leaves were removed from plants and dissected to recover larvae, 14 days after oviposition. Third- to fourth-instar larvae were removed and placed in plastic, ventilated containers (15 cm × 10 cm × 7 cm) and provided with tomato shoots with leaves, to allow for larvae to complete their development until pupation. Rearing and oviposition cages were maintained at 26 ± 1 °C, 65% RH, and at a 14 L: 10D photoperiod.

### 2.2. Entomopathogenic Fungi

Two commercial products, a wettable powder (WP) and an emulsifiable suspension (ES), of *M. anisopliae* (MET-WP and MET-ES) and *B. bassiana* (BB-WP and BB-ES), were used in the bioassays. Only the BB-WP product is currently registered for control of *T. absoluta* larvae on tomato in South Africa. The commercial EPF products with all complementary information are listed in Table 1.

### 2.3. Laboratory Dose-Response Bioassays

Laboratory bioassays were conducted to expose *T. absoluta* pupae to *B. bassiana* and *M. anisopliae* conidia, at different concentrations. The bioassays were conducted in 275 mL plastic containers (height: 9 cm, diameter: 7 cm) fitted with mesh (diameter: 3 cm) on the lids. Three holes (diameter: 4 mm) at the base of each container allowed for drainage of excess water. A commercial substrate (Seedling mix by Culterra, Johannesburg, South Africa) was autoclaved and used to fill the plastic containers to a depth of 6 cm. Distilled water (30 mL) was added as a drench to wet the substrate and initiate drainage. Pupae for the bioassays were collected daily from the rearing containers. Ten 2 to 3-day old pupae were placed onto the substrate in each container and covered with a thin layer (±3 mm) of the substrate to simulate natural pupation conditions.

Serial dilutions for each of the *M. anisopliae* and *B. bassiana* biopesticides were prepared in 1 L water, of which 40 mL was added as a drench to the respective containers. The treatments of each bioassay consisted of nine EPF concentrations per product (MET-WP: 5 × 10^8^–1.2 × 10^10^; MET-ES: 1.615 × 10^7^–2.0672 × 10^9^; BB-WP: 2.75 × 10^8^–2.2 × 10^10^; BB-ES: 5.48 × 10^5^–7.124 × 10^7^ viable conidia per litre) and a control. Distilled water was applied as the control for wettable powder products whereas, for emulsifiable suspensions, the control consisted of the products without EPF conidia (oil mixture). Each treatment was replicated four times and each container with pupae served as a replicate. Containers were maintained at 26 ± 1 °C, 65% RH with a 14 L: 10D photoperiod. The number of moths that emerged per container was recorded at three-day intervals for a period of three weeks. The mortality of pupae was calculated and expressed as a percentage. Mortality data were used to estimate the lethal concentrations for each EPF formulation.

### 2.4. Growth Tunnel Experiment

The ability of four EPF biopesticides to control *T. absoluta* pupae, when administered as a soil drench was evaluated in a greenhouse tunnel. The plant growth tunnel was covered with light defused plastic with 75% light transmission at the top and 40% white shade netting on the sides up to 1.25 m from ground level. Relative humidity and temperature were not regulated. IButton^®^ (ColdChain ThermoDynamics, Fairbridge technologies, Johannesburg, South Africa) wireless data loggers which were placed at various points throughout the plant tunnel recorded relative humidity and temperature at 30 min intervals throughout the experiment. The mean minimum and maximum percentage relative humidity (RH) and temperature during the experiment were measured as 31.45%; 16.38 °C and 82.25%; 36.61 °C with an overall mean RH of 59.80% and temperature of 24.56 °C.

Tomato seedlings were planted in 2 L pots (diameter: 16 cm) filled with a 4:1 soil:compost mixture, up to 2 cm from the rim. The soil consisted of 1.3% clay, 78% sand, 1.2% silt, with a pH of 6.49. The compost used was commercially available (Culterra, Johannesburg, South Africa). Ten 2- to 3-day-old pupae were placed onto the soil mixture in each pot and covered with a thin layer (±3 mm) of the substrate to simulate natural pupation conditions.

The four EPF products were applied as soil drench treatments at rates estimated as the number of conidia/cm^2^ which resulted in 80% mortality of the population (LC_80_) as determined in the bioassay described above. Distilled water was applied as the control treatment for wettable powder products whereas for emulsifiable suspensions the control consisted of the products without EPF conidia (oil mixture). The EPF treatments were applied at a volume of 200 mL/pot. The pots were covered with insect-proof bags made of organza material (height: 70 cm, and width: 35 cm), fitted with an elastic band at the bottom to prevent the escape of moths and to enable the recording of the numbers of eclosed moths at three-day intervals over a twelve-day period. The mortality of pupae was calculated and expressed as a percentage. Each treatment was replicated 10 times and each pot with 10 pupae served as a replicate. The experimental design was a randomised complete block design.

### 2.5. Effect on Fecundity and Fertility

The fecundity and fertility of moths of the pupae that survived the EPF treatments were determined in a laboratory bioassay, similar to that described above. However, in this bioassay, only the two EPF products (MET-ES and BB-ES) that were more effective in controlling *T. absoluta* pupae in both the laboratory bioassay and the tunnel experiment, were applied as drench treatments at the concentrations estimated as the LC_80_ per product. Six-day-old pupae were used instead of 2 to 3-day-old pupae to ensure that a sufficient number of moths emerged to successfully determine the effect of EPF biopesticides on fecundity and fertility of moths. Pupae were sexed by examining the position of genital openings [25]. Male and female pupae were exposed to EPF formulations separately.

The number of moths that emerged was recorded daily over a 7-day period. Male and female moths that emerged on the same day were paired and single pairs were placed into ventilated oviposition cages (height: 15 cm, diameter: 9 cm). A fresh tomato shoot, inserted into a plastic tube (height: 5.5 cm, diameter: 1.2 cm) filled with water, was provided as an oviposition substrate.

The pairing of moths was done according to antecedent exposure of the pupae to the respective treatments, and also included crosses between moths that emerged from treated and untreated pupae (Table 2). Fecundity and fertility of moths from the different treatment combinations were recorded daily at which time a fresh tomato shoot was provided until the females died.

Dead moths were removed from the oviposition cages to determine their infection status. These moth cadavers were individually surface-sterilized by dipping them first in 0.5% NaOCl (active chlorine) which contained 0.05% Tween 80 for 1 min, followed by 2 min in 70% ethanol, followed by two washes of 1 min each with sterile water [22]. Sterilized cadavers were incubated in Petri dishes (6 cm diameter) lined with moist filter paper and maintained at 26 ± 1 °C in total darkness for two weeks to allow for fungal growth (mycosis test). This allowed us not only to determine the incidence of moths with mycoses but also to determine whether EPFs were transferred horizontally to unexposed control moths. For treatments with *B. bassiana* conidia, external characteristically white to cream-coloured powdery sporulation on the insect integument [26] is indicative of infection, while a characteristically green-coloured sporulation indicated *M. anisopliae* infection [27].

### 2.6. Data Analysis

Abbott’s formula was used to correct the data for pupal mortality in the control treatment [28] in the laboratory bioassays and the growth tunnel experiment. Corrected mortality data from the respective laboratory dose-response bioassays were subjected to probit analysis and the relative potency ratio among responses was calculated using PoloSuite^®^ software (LeOra Software LLC, version 1.8; Northampton Northamptonshire NN1 2JL, UK). Responses were considered to be significantly different when the 95% confidence interval of the relative potency ratio did not include the value 1.

Fecundity, fertility, percentage moth emergence and corrected percentage mortality (growth tunnel) data were tested for normality (Shapiro-Wilk test) and homogeneity of variance (Levene’s test). Fecundity, percentage moth emergence, and log-transformed fertility data met these assumptions and were subsequently subjected to analyses of variance, using TIBCO Statistica™ 13.3 (TIBCO Software Inc. 2017, Palo Alto, CA, USA) [29]. Treatment means were separated using the Unequal N HSD-test at *p* = 0.05. Mycosis followed a binomial distribution (infected or not infected), and data were therefore analysed by means of binomial distribution tests. Bonferroni correction was used to adjust for multi-mean comparisons.

Corrected percentage mortality data obtained from the growth tunnel experiment were neither normally distributed nor homoscedastic, therefore the data were analysed by performing the Kruskal-Wallis test using Statistica™ 13.3 [29].

## 3. Results

The responses of *T. absoluta* pupae to the respective EPF formulations fitted the log (dose)/probit (mortality) model at *p* < 0.05 (Table 3). The slope coefficients of the four formulations ranged between 1.1 and 1.7, suggesting a homogenous response to the different mycoinsecticide.

**Figure 1:**
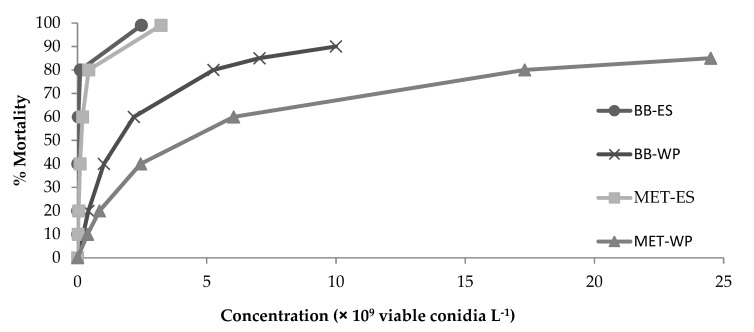
Estimated percentage mortality of *Tuta absoluta* pupae subjected to different concentrations of four entomopathogenic fungal formulations.

The estimated LC_50_ values of the respective treatments differed significantly according to the 95% confidence intervals of the relative potency ratio (*p* < 0.05) (Table 3). The oil formulations of both EPF species provided 100% control of pupae (Figure 1), and the LC_50_ values of the oil-based formulations were also significantly lower compared to the powder formulations of both products (Table 3). The BB-ES product was the most effective with an estimated LC_50_ value of 1.87 × 10^7^ viable conidia L^−1^. When the BB-WP product was applied as a drench according to the recommended field rate for foliar application against *T. absoluta* (2.2 × 10^9^ viable conidia L^−1^) to control larvae, the estimated mortality of pupae was approximately 60% (Figure 1).

High pupal mortality (68–98.8%) occurred where EPF products were applied at their respective LC_80_ concentrations under growth tunnel conditions. Pupal exposure to BB-ES resulted in significantly higher mortality (98.8%) compared to mortality of those treated with BB-WP (68.8%) (H(3) = 13.67; *p* < 0.05) (Table 3). Pupal mortality caused by *B. bassiana* (oil-based and wettable powder) and *M. anisopliae* (oil-based and wettable powder) did not differ significantly (Table 3).

Fecundity of females was significantly affected by antecedent exposure to EPFs (F = 3.02; df = 6, 61; *p* < 0.05). When males from EPF exposed pupae mated with females from *M. anisopliae* treated pupae, significantly fewer eggs were laid, with 33.0 and 35.6 eggs per female for the *B. bassiana* and *M. anisopliae* treatment respectively, compared to the fecundity of females of the control treatment (85.6 eggs per female) (Table 4). In addition, a significant reduction in fecundity was recorded in females exposed to *B. bassiana,* regardless of the EPF exposure status of the male (33.9 eggs per female) (Table 4). Mating of male moths, exposed during the pupal stage to either *B. bassiana* or *M. anisopliae,* with females from unexposed pupae did, however, not affect fecundity of females (57.7 and 57.9 eggs per female, for the respective treatments) (Table 4). Fertility was not affected by the EPF exposure status of the moths since there was no significant difference in fertility of the respective treatment pairs (F = 0.65; df = 6, 49; *p* = 0.69) (Table 4). High fertility (between 92 and 97%) was recorded for all pairs, regardless of their antecedent exposure to *B. bassiana* or *M. anisopliae* or no exposure to EPFs (Table 4).

Significantly fewer moths emerged from six-day-old pupae treated with BB-ES, compared to MET-ES treated pupae as well as from untreated control pupae, with no difference in emergence between the latter two treatments (F = 28.76; df = 2, 44; *p* < 0.001) (Table 5). Mycosis of moths from the BB-ES treatment was also significantly higher (82%) compared to the MET-ES and control treatments (F = 145.27; df = 2, 139; *p* < 0.001) (Table 5). The cadavers of moths that emerged from the uninfected pupae did not show any signs of mycosis, even after these moths mated with moths that emerged from pupae that were treated with either *B. bassiana* or *M. anisopliae* (Table 5).

## 4. Discussion

The application of synthetic insecticides forms the basis of *T. absoluta* management in Africa, although other approaches are also used [30]. It is, however, important that all life stages be targeted to control this pest. Results from this study provide evidence that EPFs can effectively kill a high percentage of *T. absoluta* pupae when applied as a soil drench. This was especially so in the case of the BB-ES formulation, which not only had the lowest estimated LC_50_ value against two-day-old pupae but proved to be fast-acting as it was also able to cause pupal mortality of approximately 70% of six-day-old pupae when they were exposed. The EPF treatments were also highly effective in the growth tunnel experiment, with 98.8% mortality of 2 to 3-day old pupae recorded. The reduced percentage of moths that emerged from pupae in the soil after BB-ES inoculation laid significantly fewer eggs. It, therefore, indicates that BB-ES inoculation to soil containing *T. absoluta* pupae may reduce the population size of subsequent generations of the pest.

For the initial laboratory bioassays, it was necessary to remove all other biotic factors in the soil to effectively evaluate the direct effects of EPF products on *T. absoluta* pupal mortality, therefore a commercially available substrate was autoclaved and used. However, in the growth tunnel experiments, tomato plants were grown in a non-sterile soil-compost mixture. Under these semi-field conditions in the growth tunnel, all EPF products exceeded the expected mortality rate of 80% when administered at their LC_80′_s as determined in laboratory bioassays, except for the BB-WP product. The higher mortality rates observed in the greenhouse tunnel experiment could therefore be ascribed to the differences in abiotic and biotic factors present in the soil such as soil texture, moisture level, temperature, and the presence of viable soil microbiota [31,32].

Females that were exposed as pupae to *B. bassiana* laid significantly fewer eggs regardless of whether or not males were treated with EPFs, indicating that *B. bassiana* infection in females significantly lowered fecundity. A reduction in fecundity was also observed for *M. anisopliae*, however only when both males and females were infected.

There was, however, no horizontal transmission of fungi between moths when fungi were acquired during the pupal stage, and mating occurred after moth eclosion from these pupae. This absence of direct horizontal transmission from infected to uninfected adults could possibly be ascribed to the adhesion of conidia to the surfaces of pupae and not to the body surfaces of moths emerging from these pupae. The importance of the availability of body surfaces for adhesion of conidia was demonstrated in *Thaumatotibia leucotreta* (Meyrick) (Lepidoptera: Tortricidae) females [33]. The latter study reported the ability of male *T. leucotreta* moths to acquire higher numbers of dry *M. anisopliae* conidia than females, to a batch of scales on the hind tibia of males, which is absent in females.

Toxicological data for fungal-based biological insecticides against pupae of *T. absoluta* was also provided by Contreras et al. [24]. The dose-responses recorded in the present study were similar to those obtained by Contreras et al. [24] who evaluated a liquid formulation of *M. anisopliae* conidia against *T. absoluta* pupae in 900 mL containers (15 × 12 × 5 cm). The estimated LC_50_ value converted to conidia/cm^2^ for MET-ES (3.59 × 10^6^ conidia/cm^2^) was, however, higher than those reported by Contreras et al. [24] for an *M. anisopliae* formulation (ranging between 1.17 and 3.0 × 10^6^ conidia/cm^2^) evaluated against different populations of *T. absoluta* in Spain. While the LC_50_ value of *B. bassiana* emulsifiable suspension (4.86 × 10^5^ conidia/cm^2^) evaluated in the current study was considerably lower than that reported by Contreras et al. [24].

BB-WP is currently the only fungus-based product registered for control of *T. absoluta* in South Africa, and it is applied as a foliar spray, specifically targeting the larval phase. This study confirmed the efficacy of the BB-WP product with *B. bassiana* strain R444 as an active ingredient against *T. absoluta.* However, to achieve *T. absoluta* pupal mortality higher than 80% with a soil application of the BB-WP product, a high dosage of at least 3.87 × 10^7^ conidia/cm^2^ will be required, which is significantly higher when compared to the other products tested.

The pupae treated in the fecundity and fertility bioassays were six days old at the time of treatment to ensure that a sufficient number of moths emerge to efficiently determine the possible effects on the abovementioned parameters. However, it also provided additional data on moth emergence when products were administered to older pupae. Bajracharya and Bhat [34] recorded an average developmental period of 7.11 days for *T. absoluta* pupae at 27 ± 2 °C. The time period available for EPFs to germinate and penetrate through the cuticles of six-day-old pupae was very short (one day). Therefore, differences in virulence between the BB-ES and MM-ES recorded in the case of older pupae could possibly be ascribed to a quicker conidial germination rate and cuticle penetration capacity of the *B. bassiana* (strain BB02) [35]. The percentage of moth emergence recorded when six-day-old pupae were treated with BB-ES and MET-ES compared to 2 to 3-day old pupae were higher as a result of a shorter period of time available to EPFs to cause infection. However, it could still result in a considerable reduction in population size even if EPF products are applied as a soil drench in a field or glasshouse of which *T. absoluta* pupae are of different ages. If BB-ES and MET-ES are applied at LC_80_ rates when most pupae are still three to four days of age, it could result in 98.8% and 90% pupal mortality with a reduction in fecundity of surviving moths of 52.6% and 50% respectively.

Previous studies on the effects of *B. bassiana* and *M. anisopliae* on *T. absoluta* larvae and eggs reported promising efficacy. Laboratory bioassays performed by Abdel-Raheem et al. [36] reported 100% mortality at 1 × 10^7^ conidia mL^−1^ when *T. absoluta* eggs were exposed to *B. bassiana* and *M. anisopliae.* İnanlı et al. [37], who dipped leaves with *T. absoluta* eggs into EPF suspensions, reported that egg mortality exceeded 90% when treated with an *M. anisopliae* product, but mortality of only 67% was recorded when eggs were treated with a *B. bassiana* product. The larval phase was also shown to be highly susceptible to isolates of both *B. bassiana* and *M. anisopliae,* resulting in mortalities exceeding 90% [22,23,38].

Soil serves as the natural ecosystem for EPFs, providing fungi with optimal moisture and temperature conditions and protection against UV radiation [31]. More importantly, soil also serves as the habitat where EPFs come into contact with the soil-dwelling life stages of insects. Consequently, the persistence of entomopathogenic fungi in the soil is a requirement for successful control. Garrido-Jurado et al. [39] reported that the availability of *B. bassiana* and *M. anisopliae* is significantly affected by soil properties, although no significant effects were recorded on the pathogenicity of EPFs. Since the rate of fungal movement through the soil profile is low, most of the available spores are retained within the superficial soil layer and persist within roots and insects after soil application [39,40]. Considering that most *T. absoluta* individuals pupate in the soil at a depth of 1–2 cm [41], there is a high probability that pupae will come into contact with conidia of entomopathogenic fungi if these are applied as drench treatments.

Plastic mulching is globally used in commercial greenhouses to increase plant productivity, conserve soil moisture, modify soil temperatures, and reduce weed pressure [42,43]. These are aspects that warrant further investigation if EPFs are considered as drench treatments. Modification of the soil microclimate caused by mulching could affect the efficacy and persistence of EPFs in the soil [44] and the resulting effects should be considered.

## 5. Conclusions

The global pest status of *T. absoluta* poses a significant threat to agricultural production and the livelihoods dependant on agricultural sustenance as is the case on the African continent. The default management strategy of applying chemical insecticides to control this pest and subsequent insecticide resistance evolution highlights the importance of using integrated management approaches in which biopesticides such as EPFs play a crucial role. This study provides evidence of the suppressive potential when administering EPF conidia as a soil drench to disrupt the life cycle of *T. absoluta* for the use in integrated pest management programs, preferably in combination with other strategies aimed at control of eggs and larvae. However, further studies need to be performed, focusing on the efficacy and persistence of EPFs as a soil drench under field conditions in soil types with different properties. Furthermore, the effectivity of EPF application via drip irrigation and the efficacy of EPFs when plastic mulching is implemented should also be investigated.

## Figures and Tables

**Table 1:** Commercial entomopathogenic fungi used and their respective target pests as per product label.

Commercial Name and Manufacturer	ACTIVE Ingredient	Experimental Name	Batch Concentration	Target Pest Registration
Metarril WP E9 Koppert SA (Pty) (Falcon Ln, Lanseria, 1739, South Africa)	*Metarhizium anisopliae* (Metsch.) Sorok., strain E9	MET-WP	2.00 × 10^9^ conidia/g	*Mahanarva fimbriolata* (Stål) (Hemiptera: Cercopidae)
Real Metarhizium 69 Real IPM (SA) Ltd. (Grabouw, 7160, South Africa)	*Metarhizium anisopliae* (Metsch.) Sorok., strain ICIPE 69	MET-ES	3.23 × 10^9^ conidia/mL	Fruit flies (Diptera) Mealybug (Hemiptera) Weevils (Coleoptera) Thrips (Thysanoptera) Whiteflies (Hemiptera)
Eco-Bb^®^ Plant Health Products (Pty) (Nottingham Road, 3280, South Africa)	*Beauveria bassiana* (Bals.) Vuill., strain R444	BB-WP	2.20 × 10^9^ conidia/g	*Spodoptera frugiperda* (J.E. Smith) (Lepidoptera: Noctuidae) *Tetranychus* spp. (Acari: Tetranychidae) *Thaumatotibia leucotreta* (Meyrick) (Lepidoptera: Tortricidae) *Tuta absoluta* (Meyrick) (Lepidoptera: Gelechiidae) Whiteflies (Hemiptera)
Real Beauveria Real IPM (SA) Ltd. (Grabouw, 7160, South Africa)	*Beauveria bassiana* (Bals.) Vuill., strain BB02	BB-ES	2.74 × 10^7^ conidia/mL	Thrips (Thysanoptera)

**Table 2:** Treatment combinations of moths that emerged from pupae exposed to emulsifiable suspensions of *Metarhizium anisopliae* and *Beauveria bassiana*.

*M. Anisopliae*-ES	*B. Bassiana*-ES
*M. anisopliae* (Male) × *M. anisopliae* (Female) MM × MF	*B. bassiana* (Male) × *B. bassiana* (Female) BM × BF
*M. anisopliae* (Male) × Control (Female) MM × CF	*B. bassiana* (Male) × Control (Female) BM × CF
Control (Male) × *M. anisopliae* (Female) CM × MF	Control (Male) × *B. bassiana* (Female) CM × BF
Control	
Control (Male) × Control (Female) CM × CF	

**Table 3:** Log-dose probit mortality data for *Tuta absoluta* pupae treated with different entomopathogenic fungal products and corrected percentage mortality for growth tunnel experiment.

Treatment	*n* ^a^ (df)	LC_50_	FL (95%) ^b^	LC_80_	FL (95%)	Slope	SE	*Χ* ^2 c^	*n* ^d^	Mean Corrected Mortality (%)
BB-ES	280 (5)	1.87 × 10^7^	1.3 × 10^7^–2.90 × 10^7^ a	1.09 × 10^8^	6.25 × 10^7^–2.50 × 10^8^	1.10	0.13	2.57	100	98.8 ± 1.3 a
MET-ES	240 (4)	1.38 × 10^8^	9.40 × 10^7^–1.88 × 10^8^ b	4.31 × 10^8^	3.15 × 10^8^–6.38 × 10^8^	1.70	0.19	2.45	100	90.0 ± 4.1 ab
MET-WP	329 (6)	3.84 × 10^9^	2.82 × 10^9^–5.5 × 10^9^ d	1.73 × 10^10^	1.05 × 10^10^–4.25 × 10^10^	1.29	0.18	5.4	100	88.8 ± 4.4 ab
BB-WP	350 (5)	1.49 × 10^9^	1.02 × 10^9^–2.0 × 10^9^ c	5.26 × 10^9^	3.9 × 10^9^–7.34 × 10^9^	1.53	0.16	3.67	100	68.8 ± 6.3 b
										H(3) = 13.67

^a^*n* = number of pupae tested; FL = fiducial limits; LCs in conidia L^−1^. ^b^ FLs within the same column followed by the same letter are not significantly different at *p* < 0.05. ^c^ Chi-square test for linearity of the dose–mortality response. ^d^ Number of pupae tested in growth tunnel experiment. Mean % corrected mortality caused by the respective treatments are not significantly different when followed by the same letter in the column (Kruskal-Wallis followed by Dunn’s multiple comparison test; *p* < 0.05).

**Table 4:** Mean fecundity and fertility (±SE) of EPF-treated and untreated *Tuta absoluta* females after mating with either EPF-treated or untreated males.

Treatment	*n*	Mean Fecundity per Female	Mean Percentage Fertility
BM × BF	10	33.00 ± 9.59 b *	93.99 ± 2.48 a *
BM × CF	9	57.67 ± 17.40 ab	92.19 ± 2.33 a
CM × BF	10	33.90 ± 9.17 b	92.40 ± 4.43 a
MM × MF	10	35.60 ± 8.06 b	96.08 ± 1.50 a
MM × CF	10	57.90 ± 10.11 ab	95.55 ± 1.63 a
CM × MF	9	67.33 ± 12.03 ab	97.26 ± 1.18 a
CM × CF	10	85.60 ± 11.41 a	96.56 ± 1.22 a

* Means within the same column followed by the same letter are not significantly different *p* < 0.05 (Unequal N HSD). *n* = Number of pairs. BM = Males treated with BB-ES formulation, BF = Females treated with BB-ES formulation, MM = Males treated with MET-ES formulation, MF = Females treated with MET-ES formulation, CM = Untreated male, CF = Untreated female.

**Table 5:** Mean percentage moth emergence and mycosis after six-day-old *Tuta absoluta* pupae were treated with EPF products.

Treatments	*n* ^a^	Mean Percentage Moth Emergence ^c^	*n* ^b^	Mean Percentage Mycosed Moths ^d^
BB-ES	157	31.60 ± 4.82 a	39	82.05 ± 6.22 a
MET-ES	189	65.82 ± 4.32 b	43	6.98 ± 3.93 b
Control	102	80.50 ± 3.20 b	60	0.00 ± 0.00 b

^a^*n* = Number of pupae tested. ^b^ *n* = Number of moths used in mycosis test. ^c^ Means within the same column followed by the same letter are not significantly different at *p* < 0.05 (Unequal HSD). ^d^ Means within the same column followed by the same letter are not significantly different at *p* < 0.05 (Binomial distribution tests, followed by Bonferroni correction).

## Data Availability

All data are provided in the paper.
